# The Spatiotemporal Neural Dynamics of Intersensory Attention Capture of Salient Stimuli: A Large-Scale Auditory-Visual Modeling Study

**DOI:** 10.3389/fncom.2022.876652

**Published:** 2022-05-12

**Authors:** Qin Liu, Antonio Ulloa, Barry Horwitz

**Affiliations:** ^1^Brain Imaging and Modeling Section, National Institute on Deafness and Other Communication Disorders, National Institutes of Health, Bethesda, MD, United States; ^2^Department of Physics, University of Maryland, College Park, College Park, MD, United States; ^3^Center for Information Technology, National Institutes of Health, Bethesda, MD, United States

**Keywords:** working memory, computational modeling, neural network, auditory object processing, fMRI, auditory-visual interaction

## Abstract

The spatiotemporal dynamics of the neural mechanisms underlying endogenous (top-down) and exogenous (bottom-up) attention, and how attention is controlled or allocated in intersensory perception are not fully understood. We investigated these issues using a biologically realistic large-scale neural network model of visual-auditory object processing of short-term memory. We modeled and incorporated into our visual-auditory object-processing model the temporally changing neuronal mechanisms for the control of endogenous and exogenous attention. The model successfully performed various bimodal working memory tasks, and produced simulated behavioral and neural results that are consistent with experimental findings. Simulated fMRI data were generated that constitute predictions that human experiments could test. Furthermore, in our visual-auditory bimodality simulations, we found that increased working memory load in one modality would reduce the distraction from the other modality, and a possible network mediating this effect is proposed based on our model.

## Introduction

Large-scale, biologically realistic neural modeling has become a critical tool in the effort to determine the mechanisms by which neural activity results in high-level cognitive processing, such as working memory. Our laboratory has investigated a number of working memory tasks in humans using functional neuroimaging and large-scale neural modeling (LSNM) in both the visual and auditory modalities. In this paper, we combine our visual and auditory models through a node representing the anterior insula to investigate the spatiotemporal dynamics of the neural mechanisms underlying endogenous (top-down) and exogenous (bottom-up) attention, and how attention is controlled or allocated in intersensory perception during several working memory tasks.

Attention is a crucial cognitive function enabling humans and other animals to select goal-relevant information from among a vast number of sensory stimuli in the environment. On the other hand, attention can also be captured by salient goal-irrelevant distractors. This mechanism, allowing us to focus on behavioral goals while staying vigilant to environmental changes, is usually described as two separate types of attention: endogenous (voluntary/goal-driven) attention and exogenous (involuntary/stimulus-driven) attention (Hopfinger and West, 2006). Endogenous attention for object features is thought to be controlled by a top-down process, starting from the frontal lobe and connecting back to early sensory areas (Kastner and Ungerleider, 2000; Koechlin et al., 2003; Bichot et al., 2015; D’Esposito and Postle, 2015; Leavitt et al., 2017; Mendoza-Halliday and Martinez-Trujillo, 2017). In contrast, exogenous attention behaves primarily in a bottom-up manner, triggered by stimuli that may be task irrelevant but salient in a given context (Yantis and Jonides, 1990; Hopfinger and West, 2006; Clapp et al., 2010; Bowling et al., 2020).

Working memory, the brain process by which selected information is temporarily stored and manipulated, relies on endogenous attention for protection from distractions (Berti and Schroger, 2003; Lorenc et al., 2021). However, working memory is not completely protected but is capable of handling unexpected and salient distractions mediated by exogenous attention (Berti et al., 2004). Early studies on the relationship between working memory and attention focused mostly on the role of endogenous attention in working memory encoding and maintenance (Baddeley, 1986, 1996). Later, some functional neuroimaging and behavioral studies showed that working memory can also control exogenous attention and reduce distractions (Berti and Schroger, 2003; Berti et al., 2004; Spinks et al., 2004; SanMiguel et al., 2008; Clapp et al., 2010). However, little is known about the brain networks mediating such effects. The aim of the present study was to investigate and propose a possible neural network mechanism of how endogenous and exogenous attention interact with each other, and how working memory controls exogenous attention switching. We restricted our analysis to the storage component of working memory (i.e., short-term memory).

We, among others, believe that computational modeling is a powerful tool for helping determine the neural mechanisms mediating cognitive functions (Horwitz et al., 1999, 2005; Deco et al., 2008; Friston, 2010; Jirsa et al., 2010; Eliasmith et al., 2012; Kriegeskorte and Diedrichsen, 2016; Bassett et al., 2018; Yang et al., 2019; Ito et al., 2020; Pulvermuller et al., 2021). With respect to working memory, Tagamets and Horwitz (1998) and Horwitz and Tagamets (1999) developed a large-scale dynamic neural model of visual object short-term memory. The model consisted of elements representing the interconnected neuronal populations comprising the cortical ventral pathway that processes primarily the features of visual objects (Ungerleider and Mishkin, 1982; Mishkin et al., 1983; Haxby et al., 1991). Later an auditory object processing model was built that functioned in an analogous fashion to the visual model (Husain et al., 2004). The two LSNMs were each designed to perform a short-term recognition memory delayed match-to-sample (DMS) task. During each trial of the task, a stimulus S1 is presented for a certain amount of time, followed by a delay period in which S1 must be kept in short-term memory. When a second stimulus (S2) is presented, the model responds as to whether S2 matches S1. Recently, the visual model was extended to be able to manage distractors and multiple objects in short-term memory (Liu et al., 2017). The extended visual model successfully performed the DMS task with distractors and Sternberg’s recognition task (Sternberg, 1969) where subjects are asked to remember a list of items and indicate whether a probe is on the list.

Here we present a simulation study of intersensory (auditory and visual) attention switching and the interaction between endogenous and exogenous attention. The term intersensory attention refers to the ability to attend to stimuli from one sensory modality while ignoring stimuli from other modalities (Keil et al., 2016). We first combine and extend the aforementioned LSNMs to incorporate “exogenous attention” (the original models already included one type of “endogenous attention”). We add a pair of modules representing “exogenous attention” for auditory and visual processing. These two modules compete with each other based on the salience of auditory and visual stimuli and assign the value of attention together with endogenous attention. Endogenous attention is set according to task specification before each simulation. Then we simulate intersensory attention allocation and various bimodal (i.e., auditory and visual) short-term memory tasks. Simulations presented below show the “working memory load effect,” i.e., higher working memory load in one modality reduces the distraction from another modality, which has been reported in a number of experimental studies (Berti and Schroger, 2003; Spinks et al., 2004; SanMiguel et al., 2008). Furthermore, we also show that higher working memory load can increase distraction from the same modality. We propose the neural mechanism that underlies intersensory attention switching and how this mechanism results in working memory load modulating attention allocation between different modalities.

## Materials and Methods

Large-scale neural network modeling aims at formulating and testing hypotheses about how the brain can carry out a specific function under investigation. Generally, the hypotheses underlying the model are instantiated in a computational framework and quantitative relationships are generated that can be explicitly compared with experimental data (Horwitz et al., 1999). Because the network paradigm now has become central in cognitive neuroscience (and especially in human studies), neural network modeling has emerged as an essential tool for interpreting neuroimaging data, and as well, integrating neuroimaging data with the other kinds of data employed by cognitive neuroscientists (Horwitz et al., 2000; Bassett et al., 2018; Kay, 2018; Naselaris et al., 2018; Pulvermuller et al., 2021).

A large assortment of neural network models has been developed, with different types aimed at addressing different questions. In two extensive reviews, Bassett and her colleagues discussed some of the various kinds of neural network models that have recently emerged (Bassett et al., 2018; Lynn and Bassett, 2019). A key distinction is made between model networks of artificial neurons and model biophysical networks (Lynn and Bassett, 2019). Deep learning networks (see LeCun et al., 2015; Saxe et al., 2021 for reviews) and recurrent neural networks (Song et al., 2016) illustrate the former, whereas a model of the ventral visual object processing pathway (Ulloa and Horwitz, 2016) and a model of visual attention (Corchs and Deco, 2002) are examples of the latter. However, this distinction is not a binary one since there has been recent work, for instance, in using deep learning models to understand the neural basis of cognition (Cichy et al., 2016; Devereux et al., 2018), and in employing recurrent network models to investigate information maintenance and manipulation in working memory (Masse et al., 2019).

The combined auditory-visual large-scale neural network model used in this paper is a biophysical cortical network model. It consists of three types of sub-models: a structural model representing the neuroanatomical relationships between modules; a functional model indicating how each basic unit of each module represents neural activity; and a hemodynamic model indicating how neural activity is converted into BOLD fMRI activity. The simulated visual inputs to the model correspond to simple shapes, and the simulated auditory inputs correspond to frequency-time patterns. The model’s outputs consist of simulated neural activity, simulated regional fMRI activity and simulated human behavior on 15 related short-term memory tasks. Because the focus of this paper is on the interaction between exogenous and endogenous attention, the auditory and visual models are connected *via* a module representing the anterior insula (see below, where details of the combined model are provided).

### The Large-Scale Neural Model Network

**FIGURE 1:**
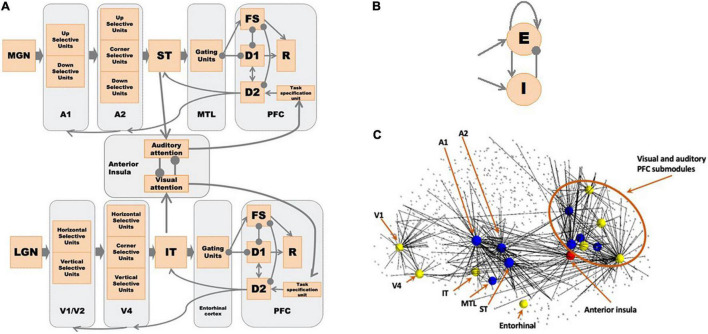
**(A)** The network diagram of the large-scale auditory-visual neural model. Arrows denote excitatory connections; lines ending in circles denote inhibitory connections (excitatory connections to inhibitory interneurons in the receiving module). The anterior insula (aINS) acts as the exogenous attention module where visual-auditory attention competition occurs. See text for details. **(B)** Structure of a Wilson-Cowan microcircuit, which can be considered as a simplified representation of a cortical column. Each microcircuit consists of an excitatory and an inhibitory element with the excitatory element corresponding to the pyramidal neuronal population in a column and the inhibitory element corresponding to the inhibitory interneurons. **(C)** Embedded model in Hagmann’s connectome (Hagmann et al., 2008). We first found hypothetical locations for our model’s regions of interest (ROIs) and the connected nodes in the connectome (small dots connected to ROIs). We embedded our model of microcircuits and network structure into the structural connectome of Hagmann et al. (2008). See Table 1 and Ulloa and Horwitz (2016) for details. The yellow nodes correspond to the visual model nodes, the blue to the auditory model nodes, and the red to the anterior insula node. The lines indicate direct connections between modeled nodes and nodes in Hagmann’s connectome.

The structural network of the combined auditory and visual model, representing the neuroanatomical relations between network modules, is shown in Figure 1A. Both the visual and auditory sub-models are organized as hierarchal networks, based on empirical data obtained from nonhuman primates and humans (Ungerleider and Mishkin, 1982; Ungerleider and Haxby, 1994; Rauschecker, 1997; Kaas and Hackett, 1999; Nourski, 2017).

**TABLE 1:** Locations of nodes in TVB chosen to host our modules.

Modules	Source	Host connectome node Talairach location
A1	Husain et al., 2004	(51 −24, 8)
A2	Husain et al., 2004	(61, −36, 12)
ST	Husain et al., 2004	(59, −20, 1)
MTL	Tanabe et al., 2005	(22, −30, −12)
V1/V2	Haxby et al., 1995	(14, −86, 7)
V4	Haxby et al., 1995	(30, −70, −7)
IT	Haxby et al., 1995	(31, −39, −6)
EC	Hagmann et al., 2008	(25, −12, −25)
FS	Location selected for illustrative purposes	(35, 19, 13), (47, 19, 9)
D1	Haxby et al., 1995; Husain et al., 2004	(51, 12, 10), (43, 29, 21)
D2	Location selected for illustrative purposes	(32, 29, 8), (42, 39, 2)
aINS	Gu et al., 2013	(48,12,4)
R	Location selected for illustrative purposes	(33, 13, 28), (29, 25, 40)

*We embedded our model into The Virtual Brain connectome based on anatomical sources listed in the table.*

As the basic units of our model, we use a variant of Wilson-Cowan units (Wilson and Cowan, 1972), which consists of one excitatory unit and one inhibitory unit (see Figure 1B). One basic unit can be considered as a simplified representation of a cortical column. Each module of the auditory subnetwork, originally developed by Husain et al. (2004), is explained in detail below. Submodules of A1 and A2 are organized as 1 × 81 arrays of basic units, and all the other modules are 9 × 9 arrays of basic units (see details below). The structure of the visual model is similar to the auditory model in many ways (one exception: the V1/V2 and V4 modules are 9 × 9 arrays of basic units); for the details of the visual model (see Tagamets and Horwitz, 1998; Ulloa and Horwitz, 2016; Liu et al., 2017). Figure 1C shows the visual and auditory models embedded in the human structural connectome provided by Hagmann et al. (2008). We used published empirical findings to posit the hypothetical brain regions of interest (ROIs) corresponding to each module in our computational model and the corresponding nodes in Hagmann’s connectome. Then we embedded our revised model of microcircuits and network structure into the connectome (see Ulloa and Horwitz, 2016, for details). We ran the simulations using our in-house simulator in parallel with Hagmann’s connectome using The Virtual Brain (TVB) software (Sanz Leon et al., 2013) (see below). Details about our simulation framework are discussed in the Appendix.

#### Module A1

**FIGURE 2:**
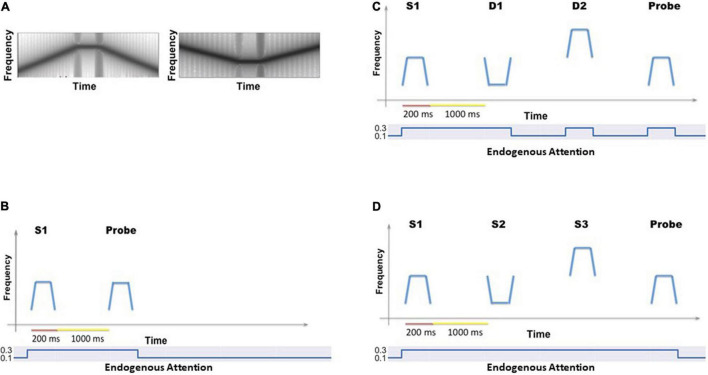
**(A)** Two examples of the auditory objects (tonal contours) we used as stimuli for the cognitive tasks. **(B)** The timeline for a single trial of the auditory delayed match-to-sample (DMS) task. The simulated subjects’ task is to identify whether the probe is a match to the first stimulus. The value of the endogenous attention parameter during each temporal epoch is shown as well. **(C)** The timeline (endogenous attention parameter value) for a single DMS trial with distractors. The simulated subjects need to ignore the intervening distractors and only respond to the probe. **(D)** The timeline (and endogenous attention parameter value) for a single trial of the auditory Sternberg’s recognition task. The simulated subjects need to remember a list of tonal contours and their task is to decide whether the probe is a match to any stimulus in the list.

The auditory model of Husain et al. (2004) that we extended here was designed to process one kind of auditory object. As pointed out by Griffiths and Warren (2004), an individual auditory object consists of sound source and sound event information (e.g., the voice of a speaker and the word produced by the speaker). In our model, the simulated auditory object of interest consists only of the sound event component—the spectrotemporal pattern of information (what we call a tonal contour; see Figure 2A). The duration of these patterns is meant to represent sounds whose duration is that of a single syllable word (∼200–300 ms).

In the model the early cortical auditory areas are combined as A1, which is analogous to the V1/V2 module in the visual model; all simulated auditory (visual) inputs enter the model *via* the A1(V1/V2) module. A1 corresponds to the core/belt area in monkeys (Rauschecker, 1998; Kaas and Hackett, 1999) and the primary auditory area in the transverse temporal gyrus in human (putative Brodmann Area 41; Talairach and Tournoux, 1988). Based on experimental evidence that the neurons in early auditory areas are responsive to the direction of frequency modulated sweeps (Mendelson and Cynader, 1985; Mendelson et al., 1993; Shamma et al., 1993; Bieser, 1998; Tian and Rauschecker, 2004; Godey et al., 2005; Kikuchi et al., 2010; Hsieh et al., 2012), module A1 was designed to consist of two types of neuronal units: upward-sweep selective and downward-sweep selective units. The two submodules are organized as 1 × 81 arrays of basic units due to the fact that in auditory cortex sounds are represented on a frequency-based, one-dimensional (tonotopic) axis (Schreiner et al., 2000; Shamma, 2001).

#### Module A2

The A2 module is designed to be a continuation of A1 and consists of three populations of units: upward sweep selective units, downward sweep selective units and contour selective units; the analogous module in the visual model is V4. The upward sweep selective units and downward sweep selective units have a longer spectrotemporal window of integration than those in A1 so that they are selective for longer frequency sweeps. The contour selective units are selective to changes in sweep direction, which are analogous with the corner selective units in the visual model. The A2 module represents the lateral belt/parabelt areas of primate auditory cortex. In experiments, parabelt neurons are found to be selective to band-pass noise stimuli and FM sounds of a certain rate and direction (Rauschecker, 1997).

#### Module ST

The third processing module of the auditory model is ST, which stands for superior temporal cortex, including superior temporal gyrus and/or sulcus and the rostral supratemporal plane. Functionally, ST is equivalent to the IT (inferior temporal) module in the visual model, and acts as a feature integrator, containing a distributed representation of the presenting stimulus (Husain et al., 2004; Hackett, 2011). This functional equivalency is supported by experimental studies that neurons in ST respond to complex features of stimuli (Kikuchi et al., 2010; Leaver and Rauschecker, 2010) and by the findings that a lesion of ST impairs auditory delayed match-to-sample performance (Colombo et al., 1996; Fritz et al., 2005).

#### Module MTL

The module MTL, a new module that we added to the original auditory model of Husain et al. (2004), represents the medial temporal lobe. It serves as a gate between ST and PFC and is incorporated so as to avoid the short-term memory representation of one stimulus being overwritten by later-arriving stimuli. MTL is analogous to the EC (entorhinal cortex) module in the visual model of Liu et al. (2017). Anatomical studies on monkeys (Munoz et al., 2009) have revealed that medial temporal lobe ablation disconnects the rostral superior temporal gyrus from its downstream targets in thalamus and frontal lobe. In our model, several groups of neurons in MTL are designed to competitively inhibit one another so that only one group of gating neurons will be activated when a stimulus comes in. Once the item is stored in this working memory buffer, an inhibitory feedback from PFC to MTL cortex will suppress the active gating neurons and will release other gating neurons so that the remaining gating neurons are ready for new stimuli. We assume that each group of MTL gating neurons can be used only once during a task trial.

#### Module PFC

The module PFC represents the prefrontal cortex in both the visual and auditory models. In the visual model, neurons in the PFC module can be delineated into four types based on experimental data acquired during a delayed response task by Funahashi et al. (1990). In our auditory model, the same four types of neuronal populations are employed analogously (Husain et al., 2004). Submodule FS contains cue-sensitive units that in general reflect the activities in the ST (IT) module. D1 and D2 submodules form the short-term memory units that excite one another during the delay period. Recently, we have built multiple sets of D1 and D2 submodules into the visual model (Liu et al., 2017) and successfully implemented tasks that hold more than one item in short-term memory; in the present study we employ the same extension in the auditory model. Submodule R serves as a response module (output). It responds when a displayed stimulus (probe) matches the cue stimulus that is being held in short-term memory. Note that we assume that there are a limited number of gating units and a similarly limited number of D1-D2 units, since empirical studies indicate that only a limited number of items can be simultaneously kept in short-term memory[e.g., the so-called 7 ± 2 (Miller, 1956); others have proposed a more limited capacity such as 3 or 4 (Cowan, 2001); however, see Ma et al., 2014 for a somewhat alternative view]. For computational simplicity, in this paper we will employ no more than three items.

#### Module aINS

The newly added aINS (anterior insula) module is represented by a pair of mutually inhibited modules. The outputs of the visual and auditory processing streams are taken as inputs for the two modules respectively and are used to generate an exogenous attention signal. The mutual inhibition between the two modules is designed to reflect the competition between modalities in salience computation. The insula area is known for its role in accumulating sensory evidence in perceptual decision-making, bottom-up saliency detection and attentional processes (Seeley et al., 2007; Menon and Uddin, 2010; Ham et al., 2013; Uddin, 2015; Lamichhane et al., 2016). In the current study, aINS processes the visual-auditory bimodality competition that leads to involuntary attention switching.

In both the visual and auditory models, a task specification module is used to provide low-level, diffuse incoming activity that can be interpreted as an attention level to the respective D2 module in the prefrontal area. We located this module arbitrarily in the superior frontal gyrus of the Virtual Brain model. The attention level/task parameter can be modulated by the outputs of the aINS module. When the attention level is low, the working memory modules are not able to hold a stimulus throughout the delay period.

The Talairach coordinates (Talairach and Tournoux, 1988) and the closest node in Hagmann’s connectome (Hagmann et al., 2008) for each of the modules discussed above (as well as for the visual model) were identified (see Table 1) based on experimental findings. As to the PFC module, which contains four submodules (FS, D1, D2, R), we used the Talairach coordinates of the prefrontal cortex in Haxby et al. (1995) for the D1 submodule in the visual model and assigned the locations of adjacent nodes for the other submodules (FS, D2, R) (see Table 1); similarly, for the PFC module in the auditory model, we used the Talairach coordinates from Husain et al. (2004) for the auditory model D1 units. This arrangement is due to the fact, as mentioned above, that the four types of neuronal populations were based on the experimental findings in monkey PFC during a visual delayed response task (Funahashi et al., 1990) and we assume that auditory working memory possesses the analogous mechanism as visual working memory. It is not known if the four neuronal types were found in separate anatomical locations in PFC or were found in the same brain region, although recent findings from Mendoza-Halliday and Martinez-Trujillo (2017) suggest that visual perceptual and mnemonic coding neurons in PFC are close (within millimeters) to one another.

See the Appendix for the mathematical aspects of the network model. All the computer code for the combined auditory-visual model can be found at https://github.com/NIDCD, as can information about how our modeling software is integrated with software for the Virtual Brain.

### Simulated Experiments

We used the combined auditory-visual model to perform several simulated experiments that included not only one stimulus, but other stimuli as well, some of which could be considered to be distractors. We created 10 “subjects” by varying interregional structural connection weights slightly through a Gaussian perturbation. For each experiment, 20 trials are implemented for each “subject.” The complete set of simulated experiments is the following:

#### Simulated Auditory Short-Term Memory Experiments:

a. Auditory delayed match-to-sample task. This experiment implemented the original delayed match-to-sample (DMS) task to demonstrate that the extended auditory model [with an added module—the MTL (medial temporal lobe), and the linkage between visual and auditory models *via* the aINS (anterior insula) module] continues to perform the DMS task and gives essentially the same results as the original model (Husain et al., 2004). One typical DMS trial consists of the presentation of a stimulus, an ensuing delay period, presentation of a probe (the same or a new stimulus); the simulated subjects need to decide whether the probe is the same as the first stimulus presented (see Figure 2B). The attention/task parameter is set to high (0.3) during a trial.

b. Auditory delayed match-to-sample task with distractors. These auditory short-term memory simulations are employed to demonstrate that the extended auditory sub-model within the combined auditory-visual model performs the analogous tasks as the visual model of Liu et al. (2017). The simulated subjects are presented with two distractors (visual or auditory) before the probe stimulus is presented (see Figure 2C). The attention/task parameter is set to high (0.3) at stimulus onset and decreases to low (0.05) following the presentation of the distractors.

c. Auditory version of Sternberg’s recognition task. An auditory variant of Sternberg’s recognition task (Sternberg, 1966, 1969) is used. On each trial of the simulation, three auditory stimuli are presented sequentially, followed by a delay period and then a probe. The subjects’ task is to decide whether the probe is a match to any of the three stimuli presented earlier (see Figure 2D). The Sternberg paradigm with visual/auditory objects has been used in many studies, and thus allows us to compare our simulated results with experimental results.

#### Simulated Visual-Auditory Bimodality Experiments

**FIGURE 3:**
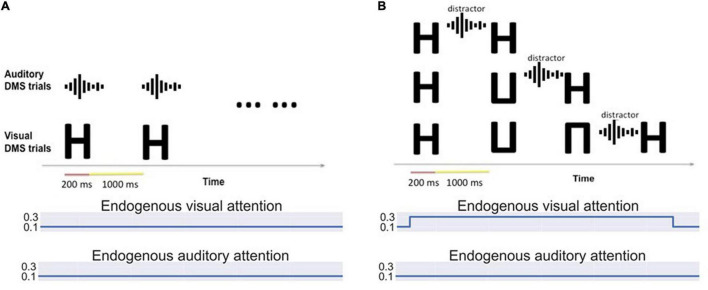
**(A)** Bimodality delayed match-to-sample task: Auditory and visual stimuli of different saliency levels are presented simultaneously. Visual and auditory endogenous attention are set to low (0.1) and exogenous attention values depend on the input saliency. The simulated subjects can choose to attend to auditory or visual stimuli based on the saliency. **(B)** Bimodality distraction task with different working memory loads. The model is asked to remember one to three visual items and decide if the final probe is a match of any stimulus in its working memory. An auditory distractor is presented before the probe.

a. Bimodality DMS task with various exogenous attention settings (see Figure 3A): A block of visual DMS trials and a block of auditory DMS trials are implemented simultaneously. The saliency of visual stimuli and auditory stimuli varies from trial to trial. The attention/task parameter assigned to each modality is determined based on the real-time output of the anterior insula module (aINS). In general, higher saliency of one stimulus will result in higher attention to the corresponding modality. Essentially, this experiment is asking a subject to perform a DMS task on the sensory modality that is more salient.

b. Bimodality distraction task with different working memory loads (see Figure 3B). The simulated subjects are asked to remember 1∼3 visual stimuli before an auditory distractor occurs. The endogenous attention is set to attend to visual stimuli.

### Simulated fMRI Experiments

Simulated fMRI signals can be calculated for each task discussed above. The direct outputs are the electrical activity of simulated neuronal units. Prior to generating fMRI BOLD time series, we first calculate the integrated synaptic activity by spatially integrating over each module and temporally over 50 ms (Horwitz and Tagamets, 1999). Using the integrated synaptic activity of select regions of interests (ROIs) as the input to the fMRI BOLD balloon model of hemodynamic response (Stephan et al., 2007; Ulloa and Horwitz, 2016), we calculate the simulated fMRI signal time-series for all our ROIs and then down-sample the time-series to correspond to a TR value of 1 s (for more mathematical and other technical details, see the Appendix; Ulloa and Horwitz, 2016).

In simulating an fMRI experiment for the aforementioned cognitive tasks, we implemented two types of design schemes: block design and event-related design. In an experiment with block design, one stimulus is followed by a 1-s delay period, and the model alternately performs a block of task trials (3 trials) and a block of control trials (3 trials). The control trials use passive perception of degraded shapes and random noises. With an event-related design, the delay period following each stimulus is extended to 20 s in order to show a more complete response curve in the BOLD signal.

## Results

### Auditory Short-Term Memory Experiments

**FIGURE 4:**
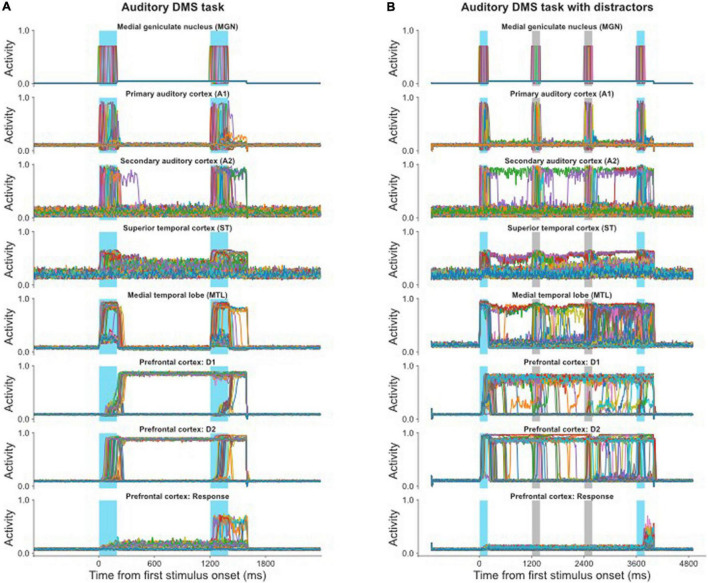
**(A)** Simulated neural activities of all the excitatory neurons in selected modules during a single auditory DMS trial (cf., Figure 2B); each neuron’s activity is represented by a different color. The blue bars indicate the presentations of stimulus and probe. Because the probe in this trial is a match with the stimulus, the Response module successfully fired during the probe presentation. **(B)** Simulated neural activities of the excitatory neurons in selected modules during a single auditory DMS trial with two intervening distractors (cf., Figure 2C). The blue bars indicate the presentations of stimulus and probe. The light gray bars indicate the presentations of distractors. The model properly avoided the distractors and responded when the probe was a match of the first stimulus.

Our first simulated experiments test whether the combined auditory-visual model can produce appropriate results (both neurally and behaviorally) for simulated auditory stimuli. Our results demonstrated that the model successfully performed the auditory DMS task (with and without distractors) and Sternberg’s recognition task with similar accuracy as the visual tasks (Liu et al., 2017; Table 2). Figure 4A shows the electrical activities of simulated neuronal units of the different modules during a DMS task. The input stimuli, represented by medial genigulate nucleus activity, are first processed by feature-selective modules in A1 and A2. A2 neurons have longer spectrotemporal windows of integration than A1 neurons and thus A2 is responsive to longer frequency sweeps. The ST module contains the distributed representation of the presenting stimulus and feeds the presentation forward to the gating module MTL and then to PFC. A working memory representation is held in the D1 and D2 modules through the delay period. The probe stimulus for Figure 4A is a match with the presented stimulus so that the R module responds.

**TABLE 2:** Model performance of the auditory tasks.

Tasks	DMS (%)	DMS w/distractors (%)	Sternberg’s task (%)
Accuracy	83.9	81.8	78.7
Standard deviation	5.71	6.73	6.05

*The model can successfully perform DMS task, DMS with distractors and Sternberg’s task with high accuracy. Values represent means and standard deviations over all simulated subjects and trials.*

The auditory model also can handle the DMS task with distractors, for which the electrical activities are illustrated in Figure 4B. The first stimulus is the target that the model needs to remember, and it is followed by two distractors. The endogenous attention/task-specification unit is set to only remember the first item. The distractors also evoke some activity in the working memory modules (D1, D2), but that activity is not strong enough to overwrite the representation of the first stimulus, so the model successfully holds its response until the matched probe appears.

In the visual model, we reported that we observed enhanced activity in the IT module during the delay period which helped short-term memory retention (Liu et al., 2017) and which was consistent with experimental findings (Fuster et al., 1982). In the current study, our modeling also displayed this type of neuronal activity in ST, as can be seen in Figure 4, and this enhanced activity has been reported in auditory experiments (Colombo et al., 1996; Scott et al., 2014).

**FIGURE 5:**
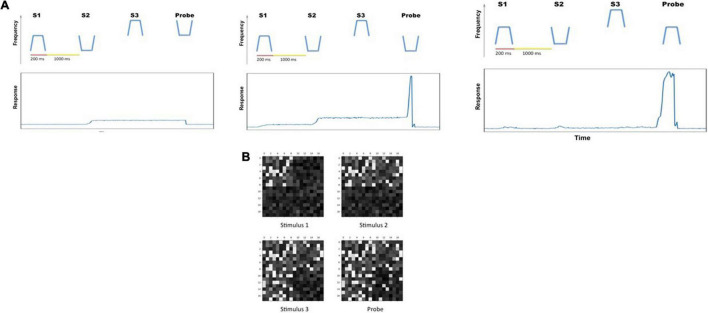
**(A)** The responses (average activity of the R units) of the model in three trials of the auditory version of Sternberg’s task. In the first trial (far left), none of the three stimuli matched the probe stimulus, and the response units showed no significant activity. In the second and third trials shown, the response module made proper responses as the probes were matches with one of the three remembered items. **(B)** Snapshots of combined working memory modules D2 during the first trial of the three trials showed in **(A)**. The snapshots are taken at the midpoint of delay interval between successive stimuli.

Figure 5 demonstrates how the model implements the auditory version of Sternberg’s recognition task and handles multiple auditory objects in short-term memory. The first three items are held in short-term memory (D1, D2), which is shown in Figure 5B, and when the probe matches any of the remembered three items the R module is activated (Figure 5A). Different groups of neurons in the gating module MTL responded to each of the stimuli and prevented the representations in working memory from overwriting one another.

### Visual-Auditory Bimodality Competition Experiments

**FIGURE 6:**
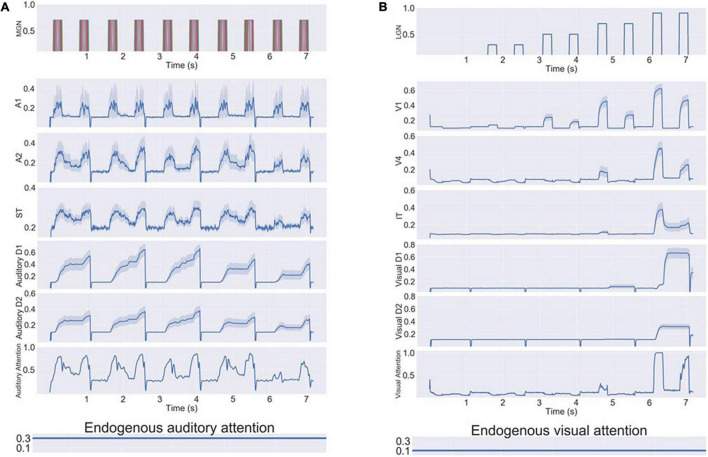
Neural activity for a series of bimodality DMS task trials with endogenous + exogenous attention. The exogenous attention assigned for visual and auditory systems (referred to as visual attention and auditory attention in the figure) is the signal outputs of the aINS submodules. The endogenous/top-down task signal is set to attend auditory stimuli and regard visual stimuli as distractors. **(A)** Neuronal activities of selected auditory modules. The auditory stimuli are stored in working memory modules (D1/D2) successfully. **(B)** Neuronal activities of selected visual modules. As the saliency level of visual stimuli increases, the exogenous attention for visual stimuli increases and the auditory attention decreases. Working memory modules D1/D2 become activated for visual distractors if they are salient compared to auditory stimuli.

Figure 6 shows the simulated intersensory attention switch caused by input saliency changes. During the simulated experiment, the attentional inputs into working memory module D2 were both the endogenous attention and the exogenous attention (output of module aINS). In the experiment shown in Figure 6, five DMS trials were implemented. The model’s endogenous attention was set to attend to auditory stimuli (the auditory attention/task parameter is set to “high”) and not to attend to visual stimuli. Thus, the model attended to auditory stimuli and treated visual stimuli as distractors in the first three DMS trials during which the saliency of auditory stimuli was higher than that of the visual stimuli. When the saliency of visual stimuli was enhanced above a certain threshold (0.8 in our modeling setting), the model started to attend to the visual stimuli and attended less to the auditory stimuli, but the model still could encode auditory stimuli into working memory.

### Working Memory Load Reduces Intersensory Distractions

**FIGURE 7:**
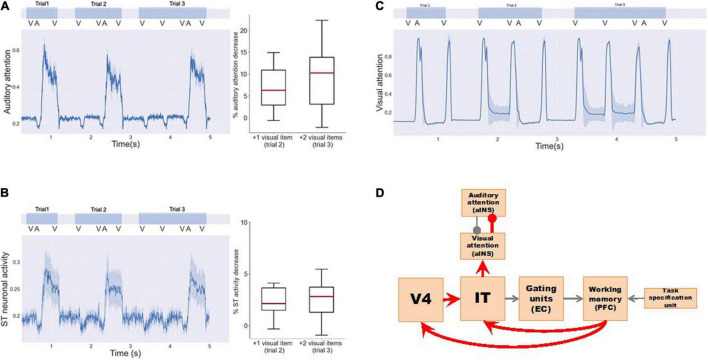
Working memory load reduces intersensory distractions. Three visual DMS trials are implemented with 1, 2, and 3 input visual stimuli, respectively. One auditory distractor is played before the final visual probe is presented. **(A)** Exogenous auditory attention in response to auditory distractors decreases when the visual working memory has more than one item. The left panel shows the average auditory attention of one simulated subject. The right panel shows the distributions of the average attention reductions of 10 simulated subjects with more visual items in working memory. One-tail t-tests were used to determine the significance of attention reductions against zero (trial 2: simulated subjects = 10, *p* = 0.0003, *t* = 4.154; trial 3: simulated subjects = 10, *p* = 0.0008, *t* = 3.727). **(B)** ST neuronal activity for auditory distractors also decreases as the visual working memory increases. The left panel shows the average ST neuronal activity of one simulated subject. The right panel shows the distributions of the average ST activity reductions of 10 simulated subjects. One-tail *t*-tests were used to determine the significance of activity reductions against zero (trial 2: simulated subjects = 10, *p* = 0.0005, *t* = 3.894; trial 3: simulated subjects = 10, *p* = 0.0003, *t* = 4.160). **(C)** Exogenous visual attention responses for visual input do not change. **(D)** Neural circuits in our model that explain the working memory load effect. Working memory loads increases exogenous visual attention *via* feedback connections to V4 and IT that in turn inhibit exogenous auditory attention to auditory distractors.

When implementing a visual DMS task with more than one item held in working memory during the delay period, the model showed a smaller response to auditory distractions (Table 3 and Figure 7). As observed in Figure 7A, exogenous auditory attention in response to auditory distractors decreases when the visual working memory has more than one item. This was demonstrated by calculating the average auditory attention values during the presentations of auditory distractors with one, two, and three items in visual working memory across 20 runs of each subject. The distributions of average auditory attention changes of 10 subjects due to more than one item in visual working memory are shown in the boxplots of Figure 7A and Table 3. The significance of auditory attention reductions with 2 and 3 items vs. with 1 item in visual working memory was tested against zero (2 items: degree of freedom = 9, *p* = 0.0003, *t* = 4.154; 3 items: degree of freedom = 9, *p* = 0.0008, *t* = 3.727). Figure 7B shows ST neuronal activity for auditory distractors is decreased as the visual working memory load is increased. Similar to auditory attention, ST activity during the presentations of auditory distractors with one, two, and three items in visual working memory was averaged across 20 runs of each subject. The distributions of average ST activity changes of 10 subjects due to more than one item in visual working memory are shown in the boxplots of Figure 7A and Table 3. The significance of ST neuronal activity reductions with 2 and 3 items in visual working memory was tested with one-tail *t*-tests against zero (2 items: simulated subjects = 10, *p* = 0.0005, *t* = 3.894; 3 items: simulated subjects = 10, *p* = 0.0003, *t* = 4.160). This phenomenon shows that the working memory formed in the model is stable and is also consistent with experimental findings that higher working memory load in one modality reduces distraction from another modality (SanMiguel et al., 2008).

**TABLE 3:** Memory load reduces intersensory distractions.

Load in visual working memory		2 items vs. 1 item (%)	3 items vs. 1 item (%)
Auditory attention changes	Mean	−6.88	−9.48
	*SD*	4.78	7.96
ST activity changes	Mean	−2.16	−2.46
	*SD*	1.54	1.84

*Visual working memory tasks with auditory distractors. With the increase of visual working memory load, the auditory attention and the activity of ST neurons for auditory distractors is reduced. Values represent means and standard deviations over all simulated subjects and trials.*

However, little is known about the underlying neural mechanism mediating these phenomena. Based on the structure of our model, we propose two possible pathways (Figure 7D) that can connect the increase in working memory load in one modality to the increase of exogenous attention in the corresponding modality, thus reducing distractions (i.e., exogenous attention) from the other competing modality: (1) D2–IT/ST–Auditory-attention. When the working memory load is high, working memory modules D1 and D2 will maintain a high activity level. Due to feedback connections from D2 to IT/ST, IT/ST and the downstream module (Auditory-attention) will exhibit increased level of activity, i.e., the exogenous attention in the corresponding modality will increase and the exogenous attention in the competing modality will decrease. (2) D2—V4/A2—IT/ST—Auditory-attention. Similar to pathway 1, the feedback connection from D2 to V4/A2, although not as strong as the connection from D2 to IT/ST, may also contribute to this working memory load effect. When the feedback connections from PFC to V4 and IT/ST in the model are removed, we no longer observed this working memory load effect. In summary, working memory load can affect intersensory neural responses through top-down feedbacks that change intersensory attention competition.

It is worth noting that the SanMiguel et al. (2008) findings hold only for intersensory tasks (i.e., tasks in which the sensory information streams are not linked). For cases where the two sensory streams are integrated (i.e., crossmodal tasks), it has been shown that increased working memory load leads to increased distraction between modalities (Michail et al., 2021).

### Simulated fMRI Experiments

The results of our simulations can be tested in humans using functional neuroimaging methods. We will illustrate this using fMRI. As discussed in section “Materials and Methods,” fMRI BOLD time series are generated for select regions of interests (ROIs) using integrated synaptic activity, and for each task we implemented the experiment using either a block design or an event-related design. The event-related scheme has longer duration delay periods than experiments using a block design. Thus, the event-related experiments can show a more complete BOLD response curve for each incoming stimulus.

**FIGURE 8:**
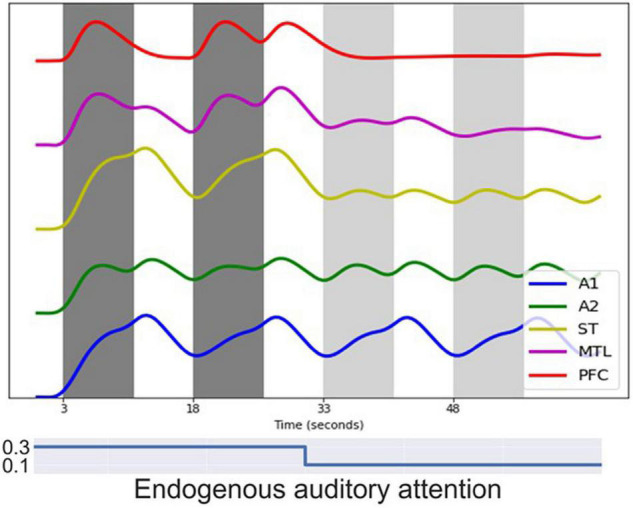
Simulated BOLD signal for a block-design auditory delayed match-to-sample (DMS) task. Each dark gray band represents a block of 3 auditory DMS trials using tonal contours and each light gray band represents a block of 3 control trials using random noise patterns. Modules representing early auditory areas A1 and A2 responded equally to DMS stimuli and noise. Higher order modules such as MTL and PFC showed larger signal changes during the DMS trials.

Figure 8 shows the simulated BOLD signal for a block-design auditory DMS task, which successfully replicates the results from Husain et al. (2004). In the simulated experiment, one block of DMS trials is followed by a block of control trials in which random noise patterns are used. Modules representing early auditory areas A1 and A2 responded equally to DMS stimuli and noises. Higher order modules such as MTL and PFC, on the other hand, show much larger signal changes, as was shown empirically by Husain et al. (2004) who employed tonal contours and auditory noise patterns.

**FIGURE 9:**
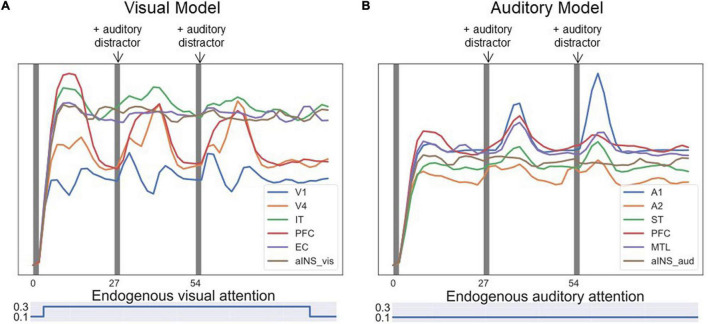
Simulated BOLD signal for an event-related visual DMS task with auditory distractors. Each of the three dark bands represent one visual DMS trial. Each trial is 2 s and the interval between trials is 25 s. During the second and the third trials, auditory distractors were played during the delay periods. **(A)** shows the BOLD signal for ROIs in the visual model and **(B)** for ROIs in the auditory model. Early auditory area A1 responded to auditory distractors but did not cause much signal changes in auditory PFC regions compared with visual PFC regions. However, the presence of auditory stimuli lowered the BOLD activity in visual PFC modules.

Figure 9 shows one event-related fMRI BOLD time-series consisting of three visual DMS trials. The probes in all three trials matched the first stimulus. During the second and the third trials, simulated auditory distractors were presented during the delay periods. Early auditory area A1 responded to auditory distractors but did not cause large signal changes in auditory PFC regions compared with visual PFC regions. The model finished all three trials correctly, but the presence of auditory stimuli lowered the BOLD activity in visual PFC modules.

**FIGURE 10:**
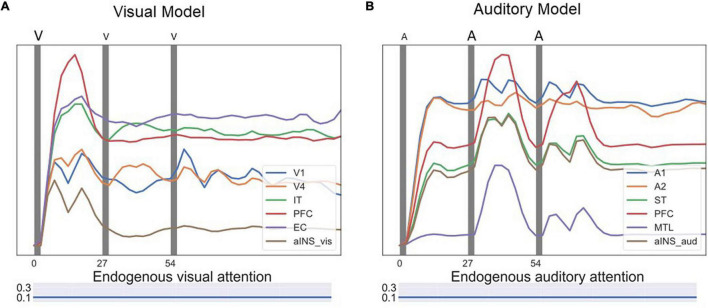
Simulated BOLD signals for an event-related visual-auditory intersensory attention allocation experiment. In the experiment, three visual DMS trials and three auditory DMS trials were presented in parallel, represented by the dark gray bands. No task instructions were given prior to the simulation, i.e., the model reacted to visual and auditory stimuli purely based on exogenous attention capture. In the first trial, the visual stimuli were more salient while in later trials auditory stimuli were more salient. The saliency levels of visual and auditory stimuli are illustrated with different font sizes above the figure. The bigger font size indicates higher saliency level. **(A)** Shows BOLD signal for ROIs in the visual model. The model attended to visual stimuli in the beginning of the test and the BOLD signal for visual PFC spiked. **(B)** Shows BOLD signal for ROIs in the auditory model. The model, after attending to visual stimuli in the beginning, switched to attending to salient auditory stimuli and the BOLD signal for the auditory PFC module increased. Endogenous attention was maintained at a low value throughout the experiment.

One experiment of visual-auditory intersensory attention allocation was also implemented. The BOLD signals of ROIs are displayed in Figure 10. No task instructions were given prior to the simulation, i.e., the endogenous attention was maintained at a low value. The model reacted to visual and auditory stimuli purely based on exogenous attention capture. The model first attended to visual stimuli as the BOLD signal for visual PFC spiked (see Figure 10A) and then switched to attend to salient auditory stimuli as the BOLD signal for auditory PFC module increased (see Figure 10B). The aINS module controls the switch by playing the role of exogenous attention.

## Discussion

In this paper we presented a large-scale biologically constrained neural network model that combines visual and auditory object processing streams. To construct this combined network model, we extended a previously developed auditory LSNM so it could handle distractors and added a module (aINS) that connected this extended auditory model with a previously established visual LSNM. The newly combined auditory-visual (AV) model can perform with high performance accuracy a variety of short-term memory tasks involving auditory and visual inputs that can include auditory or visual distractor stimuli. Multiple items can be retained in memory during a delay period. Our model, embedded in a whole brain connectome framework, generated simulated dynamic neural activity and fMRI BOLD signals in multiple brain regions.

This model was used to investigate the interaction between exogenous and endogenous attention on short-term memory performance. We simulated intersensory exogenous attention capture by presenting salient auditory distractors in a visual DMS task or salient visual distractors in an auditory task. Moreover, we simulated involuntary attention switching by presenting visual and auditory stimuli simultaneously with different saliency levels. We also investigated how working memory load in one modality could reduce exogenous attention capture by the other modality.

The AV network model used in this study was obtained by combining previously constructed visual (Tagamets and Horwitz, 1998; Horwitz and Tagamets, 1999; Ulloa and Horwitz, 2016; Liu et al., 2017) and auditory processing models (Husain et al., 2004). In our visual model, we assigned the entorhinal cortex to be the gating module between the inferior temporal area and PFC based on a series of experimental results (see Liu et al., 2017 for details). However, experimental evidence for the corresponding brain region that implements the auditory gating function is less conclusive. We based our MTL choice for the auditory gating module on a study of Munoz et al. (2009) that showed that ablation of MTL can result in disconnections between the rostral superior temporal gyrus and its downstream targets in thalamus and frontal lobe. Several cognitive tasks involving short-term memory were successfully implemented with simulated auditory stimuli. These short-term memory tasks can include auditory or visual distractor stimuli or can require that multiple items be retained in mind during a delay period. Simulated neural and fMRI activity replicated the Husain et al. results (Husain et al., 2004) when no distractors were present. The simulated neural and fMRI activity in Husain et al. (2004) model themselves were consistent with empirical data.

Neural network modeling now occupies a prominent place in neuroscience research (see Bassett et al., 2018; Yang and Wang, 2020; Pulvermuller et al., 2021 for reviews). Among these network modeling efforts are many focusing on various aspects of working memory (e.g., Tartaglia et al., 2015; Ulloa and Horwitz, 2016; Masse et al., 2019; Orhan and Ma, 2019; Mejias and Wang, 2022). Some of these employ relatively small networks (Tartaglia et al., 2015; Masse et al., 2019; Orhan and Ma, 2019), whereas others use anatomically constrained large-scale networks (Ulloa and Horwitz, 2016; Mejias and Wang, 2022). Most of these models have targeted visual processing and have attempted to infer the neural mechanisms supporting the working memory tasks under study. The AV network model used in the present study was of the anatomically constrained large-scale type.

Compared to visual processing tasks, there have been far fewer neural network modeling efforts directed at neocortical auditory processing of complex stimuli. Ulloa et al. (2008) extended the Husain et al. (2004) model to handle long-duration auditory objects (duration ∼ 1s to a few seconds; the original model dealt with object duration of less than ∼300 ms). Kumar et al. (2007) used Dynamic Causal Modeling (Friston et al., 2003) and Bayesian model selection on fMRI data to determine the effective connectivities among a network of brain regions mediating the perception of sound spectral envelope (an important feature of auditory objects). More recently, a deep learning network analysis was employed by Kell et al. (2018) to optimize a multi-layer, hierarchical network for speech and music recognition. Following early shared processing, their best performing network showed separate pathways for speech and music. Furthermore, their model performed the tasks as well as humans, and predicted fMRI voxel responses.

As noted in section “Materials and Methods,” the auditory objects that are the input to the auditory network consist of spectrotemporal patterns. However, many auditory empirical studies utilize auditory objects that also contain sound source information. For example, among the stimuli used by Leaver and Rauschecker (2010) were human speech and musical instrument sounds. Lowe et al. (2020) employed both MEG and fMRI to study categorization of human, animal and object sounds. Depending on the exact experimental design, sound source information may require long-term memory. Our current modeling framework does not implement long-term memory. Indeed, the interaction between long-term and working memory is an active area of current research (Ranganath and Blumenfeld, 2005; Eriksson et al., 2015; Beukers et al., 2021), and our future research aims to address this issue.

Our modeling of the auditory Sternberg task used a neurofunctional architecture analogous to the one used in our visual model. This is consistent with the behavioral findings of Visscher et al. (2007) who tested explicitly the similarities between visual and auditory versions of the Sternberg task.

In the simulated experiments presented in this paper, we used salience as a way to modulate exogenous attention. The salience level of one stimulus is typically detected based on the contrast between the stimulus and its surrounding environment. However, the “contrast” can be defined on different metrics, for example, the luminance of visual objects and the loudness of auditory objects, which were used in our modeling. There are other metrics based on sensory features to define a salient object, such as bright colors, fast moving stimuli in a static background, etc. An object can also be conceptually salient. A theory has been proposed that schizophrenia may arise out of the aberrant assignment of salience to external or internal objects (Kapur, 2003). In our simulation, stimuli that resulted in high working memory load may also be considered as conceptually salient, as the effect of high working memory load is similar to high endogenous attention (Posner and Cohen, 1984).

A number of brain regions are thought to be involved in the multisensory integration process (Quak et al., 2015). The perirhinal cortex has been proposed based on monkey anatomical studies (Suzuki and Amaral, 1994; Murray and Richmond, 2001; Simmons and Barsalou, 2003), whereas the left posterior superior temporal sulcus/middle temporal gyrus is suggested to be where multisensory integration takes place based on some human functional imaging findings (Calvert, 2001; Amedi et al., 2005; Beauchamp, 2005; Gilbert et al., 2013). In our study, we focused mainly on intersensory attention competition based on bottom-up salience, as opposed to multisensory integration. The anterior insula and the anterior cingulate are major components in a network that integrates sensory information from different brain regions for salience computation (Seeley et al., 2007; Sridharan et al., 2008; Menon and Uddin, 2010; Uddin, 2015; Alexander and Brown, 2019). Recent work (Ham et al., 2013; Lamichhane et al., 2016) argues that the anterior insula accumulates sensory evidence and drives anterior cingulate and salience network to make proper responses (for a review, see Uddin, 2015). Therefore, we assigned the anterior insula (aINS) as the module responsible for the exogenous attention computation and visual-auditory competition. In our model, aINS receives its inputs from IT in the visual network and ST in the auditory network and assigns values to the visual and auditory attention/task-specific unit. This arrangement has some similarities with the conceptual model of insula functioning proposed by Menon and Uddin (2010).

The model presented in this paper represents a first step in developing a neurobiological model of multisensory processing. In the present case, stimuli from the two sensory modalities are not linked. Moreover, the visual and auditory stimuli we simulated are not located in different parts of space, and thus spatial attention is not required. Nor do these visual and auditory objects correspond to well-known objects and thus do not require a long-term semantic representation. Future work will entail extending the model to incorporate long-term memory representations so that cognitive tasks such as the paired-associates task can be implemented (e.g., Smith et al., 2010; Pillai et al., 2013).

In this paper, we used our LSNM to simulate fMRI data to illustrate how our simulation model’s predictions could be tested using human neuroimaging data. Our modeling framework also can simulate EEG/MEG data. Banerjee et al. (2012a) used the visual model to simulate MEG data for a DMS task. These simulated data were employed to test the validity of a data analysis method called temporal microstructure of cortical networks (Banerjee et al., 2008, 2012b). An important future direction for our modeling effort is to enhance our framework so that high temporal resolution data such as EEG/MEG can be explored, especially in terms of frequency analysis of neural oscillations. For example, multisensory processing has been extensively investigated using such data (e.g., Keil and Senkowski, 2018).

Some caveats of our work include the following: we hypothesized that the medial temporal lobe was responsible for a gating mechanism in auditory processing, and thus the detailed location and the gating mechanism need to be confirmed by experiments. Also, the locations we chose for prefrontal nodes (D1, D2, FS, R) in the Virtual Brain are somewhat arbitrary.

The structural connectome due to Hagmann et al. (2008) that we employed here was utilized primarily so that we could compare the extended auditory model with the extended visual model of Liu et al. (2017). Its primary role was to inject neural noise into our task-based auditory-visual networks (see Ulloa and Horwitz, 2016). The Virtual Brain package allows use of other structural models, including those with higher imaging resolution (e.g., the Human Connectome Project connectome; Van Essen et al., 2013), These can be employed in future studies.

In this study, there are no explicit transmission time-delays between our model’s nodes. Such delays have been shown to play an important role in modeling resting state fMRI activity (Deco et al., 2009), and in models investigating oscillatory neural network behavior (Petkoski and Jirsa, 2019). Our models show implicit delays between nodes because of the relative slow increase of the sigmoidal activation functions. Our DMS tasks also did not require explicit and detailed temporal attention unlike other studies (e.g., Zalta et al., 2020). Transmission time-delays may be needed in future studies depending on the task design.

In summary, we have performed several auditory short-term memory tasks using an auditory large-scale neural network model, and we also simulated auditory-visual bimodality competition and intersensory attention switching by combining the auditory model with a parallel visual model. We modeled short-term auditory memory with local microcircuits (D1, D2) and a large-scale recurrent network (PFC, ST) that produced neural behaviors that matched experimental findings. For generating a brain-like environment, we embedded the model into The Virtual Brain framework. In the future the model can be extended to incorporate more brain regions and functions, such as long-term memory. Our results indicate that computational modeling can be a powerful tool for interpreting and integrating nonhuman primate electrophysiological and human neuroimaging data.

## Data Availability Statement

Publicly available datasets were analyzed in this study. These data can be found here: https://github.com/NIDCD.

## Author Contributions

QL and BH conceived, designed the study, and wrote the manuscript. QL provided software and performed data analysis. AU provided software and data analysis support. BH supervised the study, reviewed, and edited the manuscript. All authors approved the final manuscript.

## Conflict of Interest

The authors declare that the research was conducted in the absence of any commercial or financial relationships that could be construed as a potential conflict of interest.

## Publisher’s Note

All claims expressed in this article are solely those of the authors and do not necessarily represent those of their affiliated organizations, or those of the publisher, the editors and the reviewers. Any product that may be evaluated in this article, or claim that may be made by its manufacturer, is not guaranteed or endorsed by the publisher.

## Appendix

### Model Formulation

We simulated the electrical activity and fMRI activity of the model while implementing the tasks discussed in the main text using the large-scale neural modeling (LSNM) framework developed by Tagamets and Horwitz (1998), Horwitz and Tagamets (1999), Husain et al. (2004), and Liu et al. (2017). Our modeling framework is based on several critical assumptions. First, the models are constructed to perform specific tasks (e.g., the DMS task). Each submodel is designed to represent the visual/auditory object processing stream running from primary sensory cortex to prefrontal cortex. The idea is to employ neural data obtained in primate experiments, when available, to constrain the model parameters (e.g., the connection weights). For example, we used the fact that the spatial receptive field of neurons increases as one moves along the pathway from posterior to anterior cortex in the visual model; likewise, the corresponding assumption in the auditory model is that the spectrotemporal window of integration increases. The neural data generated by our model can be used to simulate neuroimaging data that in turn can be directly compared to human fMRI or PET or MEG data.

The simulated electrical activity of each E and I element of the basic unit, a modified Wilson and Cowan (1972) configuration, is determined by a sigmoidal function of the summed synaptic inputs that arrive at the unit, which corresponds to average spiking rates from single-cell recordings. This electrical activity is given by the following equations:

$$\frac{dE_{i}\left(t\right)}{dt}=\Delta \left(\frac{1}{1+e^{-K_{E}\left[w_{EE}E_{i}\left(t\right)+w_{IE}I_{i}\left(t\right)+in_{iE}\left(t\right)-\phi_{E}+N\left(t\right)\right]}}\right)-\delta E_{i}\left(t\right)$$

and

$$\frac{dI_{i}\left(t\right)}{dt}=\Delta \left(\frac{1}{1+e^{-K_{I}\left[w_{EI}E_{i}\left(t\right)+in_{iI}\left(t\right)-\phi_{I}+N\left(t\right)\right]}}\right)-\delta I_{i}\left(t\right)$$

where, Δ is the rate of change, δ is the decay rate, *K*_*E*_, *K*_*I*_ are gain constants, *w*_*EE*_, *w*_*IE*_, *w*_*EI*_ are the connectivity weights within one neuronal unit, *ϕ*_*E*_, *ϕ*_*I*_ are the input thresholds, N(t) is the noise. *in*_*iE*_*(t)*, *in*_*iI*_(*t*), are the incoming inputs from other nodes. *in*_*iE*_(*t*) is given by:

$$in_{iE}\left(t\right)=\sum_{j}w_{ji}^{E}E_{j}\left(t\right)+\sum_{j}w_{ji}^{I}I_{j}\left(t\right)+\sum_{j}c_{ji}z_{ji}^{c}C_{j}\left(t\right)$$

where, $w_{ji}^{E}$ and $w_{ji}^{I}$ are the weights for connections from the excitatory (E) and inhibitory (I) elements of *j*th LSNM unit to the excitatory element of *i*th LSNM unit, *C*_*j*_ is the electrical activity of the connectome excitatory unit *j* connected to LSNM unit *i*, and $z_{ji}^{C}$ is the connection weight. *c*_*ji*_ is a coupling term obtained by the Gaussian pseudo-random number generator of Python. *in*_*iI*_(*t*) is given by:

$$in_{iI}\left(t\right)=\sum_{k}w_{ki}^{E}E_{k}\left(t\right)+\sum_{k}w_{ki}^{I}I_{k}\left(t\right)$$

where, $w_{ki}^{E}$ and $w_{ki}^{I}$ are the weights for connections from the excitatory (E) and inhibitory (I) elements of *k*th LSNM unit to the excitatory element of *i*th LSNM unit. Each timestep corresponds to ∼5 ms.

Ulloa and Horwitz (2016) embedded the visual LSNM into a whole-brain framework using The Virtual Brain (TVB) software package (Sanz Leon et al., 2013). TVB is a simulator that combines (i) white matter structural connections among brain regions to simulate long-range connections, (ii) a neuronal population model to simulate local brain activity, and (iii) forward models that convert simulated neural activity into simulated functional neuroimaging data (i.e., fMRI or EEG/MEG). In the current study, TVB neurons provide neural noise to the embedded LSNM. The structural connectome used is due to Hagmann et al. (2008), which is composed of 998 nodes (regions of interest), and the simulated neuronal microcircuits at each TVB node are Wilson–Cowan units. The integrated synaptic activity is computed prior to computing fMRI BOLD activity by spatially integrating over each LSNM module and temporally integrating over 50 ms (Horwitz and Tagamets, 1999).

$$rSYN=\sum IN_{i}\left(t\right)$$

where, *IN*_*i*_(*t*) is the sum of absolute values of inputs to the excitatory and inhibitory elements of unit *i*, at time *t*:

$$IN_{i}\left(t\right)=w_{EE}E_{i}\left(t\right)+w_{EI}E_{i}\left(t\right)+\left|w_{IE}I_{i}\left(t\right)\right|+\sum_{k,i}w_{ki}E_{k}\left(t\right)$$

The last term is the sum of synaptic connections from all other LSNM units and connectome nodes to the *i*th unit in LSNM.

Details concerning the connectivity and the connection weights in the previous versions of the model can be found in the Tagamets and Horwitz, Husain et al., and Liu et al. papers. In brief, the connection weights are generally taken from previous papers. The connections for the new modules are hand-wired as a proof-of-hypothesis study.

### Modeling the aINS

The anterior insula is modeled as two attention modules, one for vision and one for audition, competitively inhibiting each other. The inhibition weights between the two modules are equally chosen in a way such that the two modules can be suppressed during the resting-state when only visual and auditory noises are provided and can be excited by salient visual/auditory stimuli. The choice of a specific value depends on the definition of salient stimuli.
